# Distribution and genetic diversity of multi-drug-resistant *Klebsiella pneumoniae* at the human–animal–environment interface in Pakistan

**DOI:** 10.3389/fmicb.2022.898248

**Published:** 2022-09-06

**Authors:** Bilal Aslam, Tamoor Hamid Chaudhry, Muhammad Imran Arshad, Saima Muzammil, Abu Baker Siddique, Nafeesa Yasmeen, Mohsin Khurshid, Afreenish Amir, Muhammad Salman, Muhammad Hidayat Rasool, Xueshan Xia, Zulqarnain Baloch

**Affiliations:** ^1^Department of Microbiology, Government College University Faisalabad, Faisalabad, Pakistan; ^2^National Institute of Health, Islamabad, Pakistan; ^3^Institute of Microbiology, University of Agriculture Faisalabad, Faisalabad, Pakistan; ^4^National Risk Assessment Laboratory for Antimicrobial Resistance of Animal Original Bacteria, South China Agricultural University, Guangzhou, China; ^5^Faculty of Life Science and Technology, Kunming University of Science and Technology, Kunming, China

**Keywords:** transmission, antimicrobial resistance, human-animal-environment, MLST analysis, Pakistan

## Abstract

Klebsiella pneumoniae is ubiquitous and known to be a notorious pathogen of humans, animals, and plant-based foods. *K. pneumoniae* is a recognized trafficker of antibiotic resistance genes (ARGs) between and from different ecological niches. A total of 775 samples (*n* = 775) were collected from September 2017 to August 2019 from humans, animals, and environmental sources by applying the random convenient sampling technique. A total of 120 (15.7%) samples were confirmed as *K. pneumoniae*. The distribution of *K. pneumoniae* among humans, the environment, and animals was 17.1, 12.38, and 10%, respectively. Isolates have shown significant resistance against all the subjected antibiotics agents except colistin. ARGs profiling revealed that the highest percentage prevalence (67.5%) of *bla*_*CTX*–*M*_ was estimated in the isolates, and various carbapenem resistance genes that were found in the study were *bla*_*NDM*–1_ (43.3%), *bla*_*OXA*–48_ (38%), and (1.67%) *bla*_*KPC*–2_. Overall, 21 distinct sequence types (ST) and 13 clonal complexes (CCs) were found through the multi-locus sequence typing (MLST) analysis. Taking together, the distribution of multi-drug resistance (MDR) *K. pneumoniae* clones in the  community and associated environment is alarming for the health care system of the country. Health policymakers should consider the role of all the integral parts of humans, animals, and the associated environment intently to cope with this serious public and animal health concern.

## Introduction

Antimicrobial resistance (AMR) is a mounting health concern all over the world. The global expansion of the AMR is due to the excessive and irrational use of antibiotics in clinical settings and food-producing animals. Additionally, deprived sanitation facilities, overpopulation, and inadequate sewerage disposal systems are also vital contributing factors to the dissemination of AMR (Aslam et al., 2018). Globally AMR poses huge economic losses; only in the USA losses of $35 billion have been estimated (Cecchini et al., 2015; Ventola, 2015). By the year 2050, 444 million people of the world population would be affected by resistant infections and birth rates would be reduced significantly (Bartlett et al., 2013). In the absence of new antibiotics, AR poses a severe threat to human and animal health in association with their wider environment.

The interplay between various ecologies is critically significant regarding the AMR. There is a number of associated factors among humans, animals, and the wider environment which permit not only the dissemination of various pathogens but also facilitate the transmission of mobile genetic elements carrying the antibiotic resistance genes (ARGs). To develop pragmatic and useful predictions regarding AMR, we need to have adequate and comprehensive data about the dynamics of different antibiotics, multidrug-resistant (MDR) pathogens, and the presence of various resistance determinants in different hosts and the wide-ranging environment (Woolhouse et al., 2015).

The ecological range of *Klebsiella pneumoniae* in various hosts and environments has been well documented (Wyres and Holt, 2016). Since the 1970s, the ubiquitous distribution of *K. pneumoniae* has been reported, and it is known to be a contaminant of animal- and plant-based foods (Davis and Price, 2016). *K. pneumoniae* is a pathogenic Gram-negative bacterium, a member of the Enterobacterales. World Health Organization (WHO) declared the carbapenem-resistant Enterobacterales (CRE) as a health threat of critical importance (Ventola, 2015).

Population genomics of *K. pneumoniae* revealed that it is characterized by mass scale diversified and deep-branching clonal groups. There are approximately 5,500 genes present in *K. pneumoniae* genomes, 2,000 genes shared amongst different strains, and approximately, 3,500 genes that have a major part of the AMR genes, which are collected from a wide-ranging pool (≥ 30,000) of protein-coding sequences. Only a few clonal groups from universally distributed *K. pneumoniae* proved dexterous to acquire ARGs (Wyres and Holt, 2016).

In Pakistan, available data are not significant, and routine surveillance at the national level has been overlooked. Lately, we reported the emergence of MDR *K. pneumoniae* in the hospital and veterinary settings (Chaudhry et al., 2019; Aslam et al., 2020; Chaudhry et al., 2020). Herein, the present study was designed to find out the distribution and genetic diversity of *K. pneumoniae* in humans, animals, and their associated environments.

## Materials and methods

### Sample collection

Using a random convenient sampling technique, a total of (*n* = 775) samples were collected by following standard aseptic conditions and microbiological procedures. In detail, clinical samples (*n* = 525) including urine samples (*n* = 122), tracheal aspirates (*n* = 89), wounds and pus (*n* = 158), and blood (*n* = 156) were collected from different hospitals. The environmental samples (*n* = 210), including wastewater (*n* = 39), were taken from hospital effluents and nearby communities, and other samples were collected from operation theater waste (*n* = 31), hospital sludge (*n* = 23), and ward waste (*n* = 19), and abattoir/farm wastewater (*n* = 65) and veterinary sludge/waste (*n* = 33) were collected from healthy and infected animals at public and private dairy farms and slaughterhouses. Veterinary source samples (*n* = 40) include milk (*n* = 18) and fecal samples (*n* = 22) from the animals.

### Isolation and identification of *Klebsiella pneumoniae*

In brief, some of the samples including fecal swabs, abattoir samples, and ward and operation theater waste samples were dispensed into sterilized glass tubes containing 1 mL PBS followed by streaking on nutrient agar plates; on the other hand, the remaining samples including wastewater, clinical specimens, milk, and sludge samples were streaked directly on nutrient agar plates. Plates were incubated for 24 h at 37°C. Afterward, selective media named HiChrom Klebsiella Selective agar (Himedia^®^) was used for the isolation of *K. pneumoniae*. Biochemical characterization of the isolates was done by API 20E Kit and VITEK identification system (bioMérieux, France). For molecular identification of the isolates, 16S rDNA PCR was performed. A total of 35 PCR cycles were performed for the amplification after initial denaturation at 95°C for 3 min. The cycle specifications include: denaturation at 95°C for 35 s, annealing at 50°C for 30 s, extension at 72°C for 60 s, and a final extension was done at 72°C for 5 min. Subsequently, to visualize the amplified PCR product agarose (Thermo Fisher Scientific, United States) gel electrophoresis was performed and gel documentation system (Bio-Rad, United States) was used to read the PCR products.

### Antibiotic susceptibility testing

For antibiotic resistance (AR) profiling of the isolates, different antibiotics with CLSI recommended concentrations were used (CLSI, 2018). Antibiotics used in the study were ampicillin 10 μg and piperacillin 100 μg, cefuroxime 30 μg, cefixime 5 μg, ceftriaxone 30 μg, and cefepime 30 μg, meropenem 10 μg, ciprofloxacin 5 μg, tetracycline 30 μg, and minocycline 30 μg, trimethoprim-sulfamethoxazole 1.25/23.75 μg, colistin 10 μg, and tigecycline 15 μg. *Escherichia coli* (ATCC-25922) was used as a quality control strain.

In addition to the disc diffusion method, the minimum inhibitory concentration (MIC) of all the antibiotics in the study was determined through broth microdilution assay as described previously (Chaudhry et al., 2020). Precisely, freshly grown *K. pneumoniae* was used to make 0.5 McFarland standards. Different ranges (0.06–256 μg/mL) of antibiotics dilutions were made. Micro-titration plate wells having growth were used to take inoculum, which was streaked on Petri plates containing nutrient agar and incubated for 24 h at 37°C. Results were interpreted according to the CLSI, 2018 recommendations.

### Detection of antibiotic resistance genes

DNA extraction was done by using a DNA Extraction kit (Qiagen™, Hilden, Germany). DNA quantification was performed through NanoDrop 2000 (Thermo Scientific™, United States), and DNA ≥ 60 ng/μl concentration was used for the further experiment. Extracted DNA from freshly grown isolates was used for genetic screening and the detection of β-lactamase genes, including ESBL genes (*bla*_*TEM*_, *bla*_*SHV*_, *bla*_*CTX*–*M*–1_, *bla*_*CTX*–*M*–2_, *bla*_*CTX*–*M*–9_, *bla*_*CTX*–*M*–14_, and *PAN*_*CTX*–*M*_), carbapenemases genes (*bla*_*KPC*_, *bla*_*IMP*_, *bla*_*VIM*_, *bla*_*GIM*_, *bla*_*NDM*–1_, and *bla*_*OXA*–48_), fluoroquinolones (*qnr*A, *qnr*B, *qnr*S, *par*C, *gyr*A, and *gyr*B), tetracycline (*tet*A and *tet*B), and sulfonamides (*sul*1 and *sul*2) genes. The amplicons were sent for Sanger sequencing to Macrogen (Seoul, South Korea), and the obtained sequences were verified using the NCBI BLAST tool.

### Multi-locus sequence typing

The MLST was carried out by following the Pasteur MLST scheme by the amplification of seven housekeeping genes of *K. pneumoniae.* The PCR was carried out by making up a 50 μl reaction mixture that comprises 25 μl of 2X PCR Master Mix (Thermo-Scientific, United States), and each primer (10 μM) was 1 and 2 μl sample DNA. The following PCR cycle conditions were set: initial denaturation at 94°C for 2 min and then 35 cycles of initial denaturation at 94°C for 20 s. The annealing temperature for all the genes were set at 50°C, except for *tonB* (45°C) and *gapA* (60°C), for 30 s and extension at 72°C for 30 s, and at the end, a final extension at 72°C for 5 min was performed in PCR Thermal Cycler (Bio-Rad Inc., United States) (Diancourt et al., 2005). The amplified product of respective genes was analyzed on ethidium bromide-stained 1% agarose gel, the band size was assessed by GeneRuler 1 kb plus DNA ladder (Thermo-Scientific™, United States), and the snapshots were taken by gel documentation system (ChemiDoc™ XRS + System, Bio-Rad, United States). The genes were sequenced with the same forward and reverse sequencing primers from Macrogen™. For the determination of sequence types (ST), the MLST database for *K. pneumoniae* was referred according to the pasture scheme.^1^

### Conjugation assay

To assess the transferability of *K. pneumoniae* isolates, conjugation assay was performed. Isolates of the study were kept as a donor while *E. coli* cells (Thermo Fisher^®^, United States) were used as the recipient bacterial cells. Confirmation of the transconjugants was done by streaking on Luria-Bertani (LB) agar supplemented with meropenem (MEM), imipenem (IMP), and ertapenem (ETP). Colony PCR of the transconjugants was also performed by using specific primers of *bla*_*NDM*–1_ *bla*_*KPC*_ and *bla*_*OXA*–48_. MICs of the transconjugants were determined according to CLSI (2018) guidelines.

## Results

### Distribution of *Klebsiella pneumoniae* among different sample sources

Overall, out of (*n* = 775) the collected samples, a total of 120 isolates were confirmed as *K. pneumoniae.* From *n* = 525 clinical samples, a total of 90 isolates were *K. pneumoniae*, while from (*n* = 210) environmental samples, a total of 26 isolates were *K. pneumoniae*, and out of veterinary samples (*n* = 40), a total of 4 isolates were confirmed *K. pneumoniae* (Table 1).

**TABLE 1:** Details of all the collected samples and percentage prevalence of *K. pneumoniae* from various sample sources.

Sample type	Number of isolates(n)	Prevalence percentage (%)
**Human**		
Urine (*n* = 122)	13	10.6
Sputum (*n* = 89)	21	23.6
Wounds and Pus (*n* = 158)	21	13.3
Blood (*n* = 156)	35	22.4
**Total (*n* = 525)**	**90**	**17.1**
Chi sq-stat = 10.950; alph- chi-sq critical = 7.8147; *p*-value chi sq test = 0.0120		
**Environment**		
Ward waste (*n* = 19)	3	16
Operation theater waste (*n* = 31)	1	3
Wastewater (*n* = 39)	9	23
Hospital sludge (*n* = 23)	4	17
Abattoir/wastewater (*n* = 65)	7	11
Veterinary sludge/waste (*n* = 33)	2	6
**Total (*n* = 210)**	**26**	**12.38**
Chi sq-stat = 8.615; alph- chi-sq critical = 11.070; *p*-value chi sq test = 0.125		
**Animals**		
Milk (*n* = 18)	1	5
Fecal samples (*n* = 22)	3	13.6
**Total (*n* = 40)**	**4**	**10**
Chi sq-stat = 0.7182; alph- chi-sq critical = 3.8414; *p*-value chi sq test = 0.3967		

### Resistance profiling

All isolates (100%; *P* < 0.05) were found resistant to ampicillin and piperacillin, while 114 (95.0%; *P* < 0.05) isolates were found resistant to cefuroxime. Moreover, 97 (80.8%; *P* < 0.05) isolates were found resistant to the third generation cephalosporins named cefixime and ceftriaxone, while 71 (59.2%; *P* < 0.05) isolates were found resistant to cefepime. The resistance to carbapenems (meropenem) was found in 39 (32.5%) isolates. Moreover, 87 (72.5%) and 45 (37.5%) isolates were found resistant to moxifloxacin and ciprofloxacin, respectively, while 65 (54.2%; *P* < 0.05) and 72 (60.0%; *P* < 0.05) isolates were resistant to minocycline and tetracycline, respectively. Although 65 (54.2%; *P* < 0.05) isolates were resistant to cotrimoxazole and 18 (15.0%) isolates were found resistant to chloramphenicol, only 01 isolate was found resistant to tigecycline. Fortunately, all the isolates were found susceptible to colistin (Table 2).

**TABLE 2:** Antibiotic resistance profile of indigenous *K. pneumoniae* isolates (*n* = 120) against all the antibiotics used in the study.

Antibiotics	Resistance	Intermediate	Susceptible
Ampicillin	120 (100%)	0 (0.0%)	0 (0.0%)
Piperacillin	120 (100%)	0 (0.0%)	0 (0.0%)
Cefuroxime	114 (95.0%)	0 (0.0%)	6 (5.0%)
Cefixime	97 (80.8%)	2 (1.7%)	21 (17.5%)
Ceftriaxone	97 (80.8%)	2 (1.7%)	21 (17.5%)
Cefepime	71 (59.2%)	0 (0.0%)	49 (40.8%)
Meropenem	39 (32.5%)	4 (3.3%)	77 (64.2%)
Ciprofloxacin	45 (37.5%)	5 (4.2%)	70 (58.3%)
Moxifloxacin	87 (72.5%)	0 (0.0%)	33 (27.5%)
Minocycline	65 (54.2%)	4 (3.3%)	51 (42.5%)
Tetracyclines	72 (60.0%)	3 (2.5%)	45 (37.5%)
Tigecycline	1 (0.8%)	3 (2.5%)	116 (96.7%)
Chloramphenicol	18 (15.0%)	0 (0.0%)	102 (85.0%)
Colistin	0 (0.0%)	0 (0.0%)	120 (100.0%)
Cotrimoxazole	65 (54.2%)	0 (0.0%)	55 (45.8%)

### Detection of antibiotic resistance genes

Molecular AR profiling of all (120) the isolates revealed that *bla*_*SHV*_ *was* detected in 92.5% followed by *bla*_*CTX*–*M*_ (67.5%; *P* < 0.05), while other ARGs which were detected in the isolates showed the percentage prevalence as follows: *bla*_*TEM*_ (57.5%; *P* < 0.05), *bla*_*CTX*–*M*–1_ (64%; *P* < 0.05), *bla*_*CTX*–*M*–2_, (11.6%), *bla*_*CTX*–*M*–9_ (27.5%), *bla*_*CTX*–*M*–14_ (49%), *bla*_*NDM*–1_ (43.3%), and *bla*_*OXA*–48_ (38%), and all the isolates were negative for *bla*_*VIM*_, *bla*_*GIM*_, and *bla*_*IMP*_. One of the most significant findings of the study was the detection of the *bla*_*KPC*–2_ gene among the 02 isolates i.e., one isolate from a clinical source and one from the veterinary source were found positive for *bla*_*KPC*–2_ (Table 3). In addition to the ESBL and carbapenemases genes, various other ARGs which were included in the study showed the following distribution patterns among *K. pneumoniae* isolates: *tet*A (40%), *tet*B (45%), *qnr*S (36.6%), *qnr*B (44%), *sul*1 (39%), and *sul*2 (44%), respectively.

**TABLE 3:** Prevalence of all beta-lactamases genes found in isolates from different sources.

Beta-lactamases genes	Sequence	Reference	All Isolates n (%)	Human n (%)	Environment n (%)	Veterinary n (%)
***bla*** **_*TEM*_**	F-TCAACATTTCCGTGTCG R-CTGACAGTTACCAATGCTTA	(Chaudhry et al., 2020)	69 (58%)	47 (52%)	9 (53%)	13 (100%)
***bla*** **_*SHV*_**	F-ATGCGTTATATTCGCCTGTG R-AGATAAATCACCACAATGCGC		119 (99%)	88 (98%)	17 (100%)	13 (100%)
***bla*** **_*CTX*–*M*–1_**	F-GTTTCCGCTATTACAAACCGTTG R-GCCCATGGTTAAAAAATCACTGC		77 (64%)	54 (60%)	12 (71%)	11 (85%)
***bla*** **_*CTX*–*M*–2_**	F-TGGAAGCCCTGGAGAAAAGT R-CTTATCGCTCTCGCTCTGTT		14 (12%)	5 (6%)	7 (41%)	2 (15%)
***bla*** **_*CTX*–*M*–9_**	F-ATGGTGACAAAGAGAGTGCA R-CCCTTCGGCGATGATTCTC		33 (28%)	22 (24%)	7 (41%)	4 (31%)
***bla*** **_*CTX*–*M*–14_**	F-GAGAGTGCAACGGATGATG R-TGCGGCTGGGTAAAATAG		59 (49%)	41 (46%)	7 (41%)	11 (85%)
**PAN*bla*_*CTX*–*M*_**	F- GGATATCGTTGGTGGTGCCATA R-TTTGCGATGTGCAGTACCAGTAA		81 (68%)	55 (61%)	13 (76%)	13 (100%)
***bla*** **_*KPC*–2_**	F-TGCAGAGCCCAGTGTCAGTTT R-CGCTCTATCGGCGATACCA		2 (2%)	1 (1%)	0 (0%)	1 (8%)
***bla*** **_*IMP*_**	F-GGAATAGAGTGGCTTAATTCTC R-CCAAACCACTACGTTATC		0 (0%)	0 (0%)	0 (0%)	0 (0%)
***bla*** **_*VIM*_**	F-GATGGTGTTTGGTCGCATA R-CGAATGCGCAGCACCAG		0 (0%)	0 (0%)	0 (0%)	0 (0%)
***bla*** **_*GIM*_**	F-TCGACACACCTTGGTCTGAA R-AACTTCCAACTTTGCCATGC		0 (0%)	0 (0%)	0 (0%)	0 (0%)
***bla*** **_*NDM*–1_**	F-TGCCCAATATTATGCACCCGG R-CGAAACCCGGCATGTCGAGA		52 (43%)	31 (34%)	9 (53%)	12 (92%)
***bla*** **_*OXA*–48_**	F-TTGGTGGCATCGATTATCGG R-GAGCACTTCTTTTGTGATGGC		46 (38%)	29 (32%)	6 (35%)	11 (85%)

Selected isolates after molecular identification and ARG’s detection were subjected to sequencing. Obtained sequences were used for BLAST analysis and submitted for NCBI GenBank accession numbers, which include MK620996.1, MK620997.1, MK620998.1, MK620999.1, MK621000.1, MK621001.1, MK621002.1, MK585512.1, MK585513.1, and MK458727.1.

### Distribution of sequence types

**FIGURE 1:**
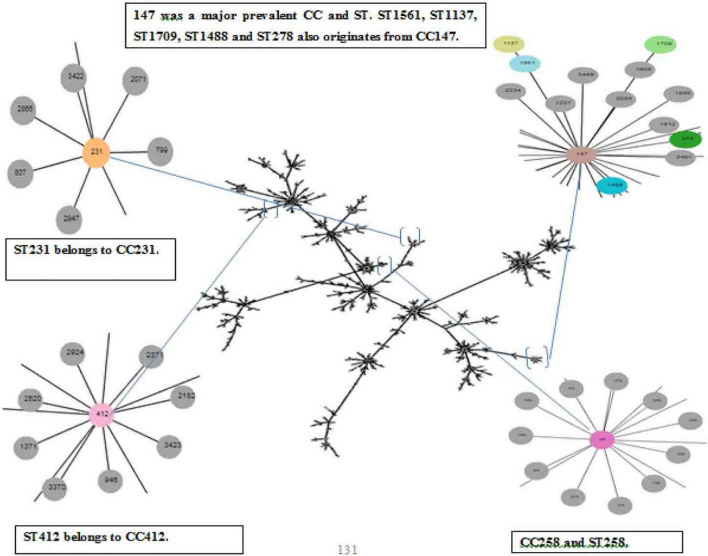
eBURST analysis (Phyloviz) of Klebsiella pneumoniae representing the STs and CCs, Partial snapshots of branches magnified and highlighted each CC and ST found in the study.

**FIGURE 2:**
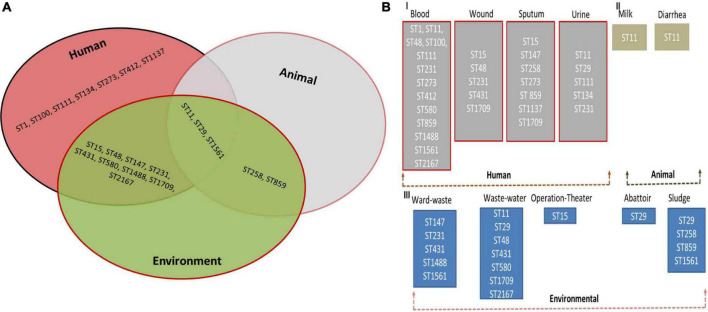
**(A)** Distribution of multilocus sequence types (STs) of Klebsiella pneumoniae in Human, Animal, and Environment. **(B)** Prevalence of multilocus sequence types (STs) in blood, wound, sputum, urine (Human) **(I)**, milk and fecal (Animal) **(II)** and ward-waste, wastewater, operation-theater, Abattoir, and sludge (Environment) **(III)**.

The panel of 120 *K. pneumoniae* isolates revealed 21 distinct sequence types (ST), while a total of 13 clonal complexes (CCs) were observed (Figure 1). The distribution of STs in humans, animals, and the environment has been shown in Figure 2A. Various STs found from clinical samples were ST1, ST100, ST111, ST134, ST274, and ST412 (Figure 2BI). ST11 was a single ST found in veterinary (animal) sources (milk and diarrhea, Figure 2BII), whereas different STs which were found in the isolates of environmental sources were also observed in the isolates of clinical sources, these STs were as follows: ST48, ST15, ST231, CC147, ST431, ST580, ST859, ST1137, ST1488, ST1561, ST1709, and ST2167 (Figure 2BIII). While all the STs observed in the isolates of veterinary and veterinary environment sources were also observed in the isolates of clinical sources, these STs were as follows: ST11, ST29, and ST258 (Figure 2A).

Thirteen isolates, comprising seven isolates from clinical and six isolates from veterinary sources, matched ST11 (*P* < 0.05). ST48 was the second most abundant sequence type corresponding to the 12 isolates and ST48 (*P* < 0.05) was the most commonly found ST in the human sources as the 11 clinical isolates. Both dominant STs were classified as MDR with high resistance profile against majorly used antibiotics. CC231 (ST231) was observed in 09 isolates. ST431 and ST231 STs were different from each other by single-locus variant (SLV), and ST15 was found to be the originator genotype of clonal complex CC15. Furthermore, the four diverse STs of CC147 including ST1137, ST1488, ST1561, and ST580 were also found to have double locus variants (DLV) to each other.

Another ST258 corresponded to the 1 + 1 isolate equally from sputum and veterinary sludge, which was designated as XDR with high resistance profile against 13 antibiotics and showed a marked presence of *bla*_*kpc*–2_, *bla*_*NDM*–1_, and *bla*_*OXA*–48_. The presence of these carbapenems encoding genes was also evident in ST11, which is a SLV of ST258, but both belong to different CCs. There are few isolates of human and environmental origins that matched with ST1709 and ST2167, which were quite dissimilar to each other in allelic profiles, and all these isolates were found susceptible to all the antibiotics used in the study. Details of STs harboring carbapenem resistance genes are given in Table 4.

**TABLE 4:** The genetic diversity of indigenous *K. pneumoniae* isolates from different sources.

Sequence type (Pasteur Scheme)	Clonal complex	Allelic profile *(gapA, infB, mdh, pgi, phoE, rpoB, tonB*)	No. of isolates	Sources	No. of resistant antibiotics	MDR/XDR	Acquired carbapenem resistance mechanism
ST1	1	4 + 4 + 1 + 1 + 7 + 4 + 10	2	Clinical (2)	11	MDR	
ST11	11	3 + 3 + 1 + 1 + 1 + 1 + 4	13	Clinical (7) veterinary (6)	10	MDR	*bla*_*NDM*–1_ *bla*_*OXA*–48_
ST15	15	1 + 1 + 1 + 1 + 1 + 1 + 1	9	Clinical (8) environmental (1)	10	MDR	–
ST29	29	2 + 3 + 2 + 2 + 6 + 4 + 4	9	Clinical (3) veterinary (6)	8	MDR	–
ST48	48	2 + 5 + 2 + 2 + 7 + 1 + 10	12	Clinical (11) environmental (1)	8	MDR	–
ST100	101	2 + 6 + 5 + 5 + 4 + 4 + 6	1	Clinical (1)	4	MDR	–
ST111	111	2 + 1 + 5 + 1 + 17 + 4 + 42	7	Clinical (7)	11–12	XDR (1) MDR (6)	*bla*_*NDM*–1_ *bla*_*OXA*–48_
ST134	292	3 + 1 + 2 + 1 + 1 + 1 + 4	3	Clinical (3)	8	MDR	–
ST147	147	3 + 4 + 6 + 1 + 7 + 4 + 38	3	Clinical (2) environmental (1)	10	MDR	*bla*_*OXA*–48_
ST231	231	2 + 6 + 1 + 3 + 26 + 1 + 77	10	Clinical (9) Environmental (1)	8–9	MDR	*bla*_*OXA*–48_
ST258	258	3 + 3 + 1 + 1 + 1 + 1 + 79	2	Clinical (1) Veterinary (1)	13	XDR	*bla*_*NDM*–1_ *bla*_*OXA*–48_
ST273	147	3 + 4 + 6 + 1 + 7 + 4 + 4	8	Clinical (8)	10	MDR	*bla*_*OXA*–48_
ST412	412	2 + 1 + 2 + 1 + 9 + 1 + 112	2	Clinical (2)	9	MDR	–
ST431	15	2 + 1 + 1 + 1 + 1 + 1 + 1	3	Clinical (2) environmental (1)	9	MDR	–
ST580	147	3 + 4 + 6 + 1 + 9 + 4 + 38	4	Clinical (2) environmental (2)	4	MDR	–
ST859	11	3 + 3 + 1 + 1 + 1 + 1 + 1	6	Clinical (5) environmental (1)	9	MDR	*bla*_*NDM*–1_
ST1137	147	3 + 4 + 6 + 4 + 7 + 4 + 38	6	Clinical (5) environmental (1)	5	MDR	–
ST1488	147	3 + 4 + 2 + 1 + 7 + 4 + 38	4	Clinical (3) environmental (1)	4	MDR	–
ST1561	147	3 + 4 + 6 + 1 + 7 + 1 + 38	5	Clinical (2) environmental (3)	6	MDR	–
ST1709	147	3 + 1 + 1 + 1 + 7 + 4 + 38	8	Clinical (6) environmental (2)	3	MDR	–
ST2167	37	2 + 9 + 2 + 1 + 13 + 1 + 35	3	Clinical (1) environmental (2)	5	MDR	–

### Conjugation validation

All the selected isolates displayed transferability, and colonies of the transconjugants were observed on LB agar plates containing MEM, IMP, and ETP. The MIC_90_ of these antibiotics (MEM, IMP, and ETP) against transconjugants were 32, 16, and 16 μg/mL, respectively. Various carbapenemases genes (*bla*_*NDM*–1_ *bla*_*KPC*_ and *bla*_*OXA*–48_) were detected by colony PCR, which confirmed the transconjugants.

## Discussion

The mounting crisis of AR is a global challenge that is affecting humans as well as animals in association with the wider environment. Irrational use of antibiotics in health care settings, veterinary and agricultural practices is a substantial contributing factor in the global expansion of AR. Moreover, poor sanitation and disposal systems associated with massive populations have a threatening impact on the dissemination of AR to the community (Adnan et al., 2017; Aslam et al., 2018; Baloch et al., 2018). Pakistan is one of the leading countries in the production of animal products, and the use of antibiotics in animals and veterinary practices is a routine matter in the country. Despite intense modernized farming, Pakistan still lacks sufficient data on the use and estimation of antibiotics in animals (Hassan Ali et al., 2010; Idrees et al., 2011; Khan et al., 2013; Adnan et al., 2017; Mohsin et al., 2017; Mohsin, 2019).

The findings of the present study showed that overall, 120 (15.7%) were confirmed *K. pneumoniae* isolated from various sources of humans, animals, and the environment (Figure 2A). It has been well established that *K. pneumoniae* can survive in different environments because of its large genome size (5.7 Mbps with 5,455 protein) than *E. coli* (5.1 Mbps with 4,915 genes) (Sudarwanto et al., 2015). A total of 90 (17.42%) *K. pneumoniae* isolates were obtained from clinical samples. Isolation of *K. pneumoniae* has been reported from various clinical specimens across Pakistan. Most of the isolates of the present study were obtained from respiratory samples, i.e., sputum (23.6%), blood samples (22.4%) followed by pus samples (13.3%) collected from various wounds and surgical sites, and urine samples (14.4%) collected from UTI-infected male and female patients. The is important and suggested that it may lead to hospital-acquired infection or put patients at risk of ventilator-associated pneumonia and bacteremia due to *K. pneumoniae*. Same findings have been reported from Aga Khan University Hospital, Karachi. Pakistan, where they also found comparable percentage prevalence in various sample sources, particularly blood samples (Khan et al., 2010b). The same study has also been reported from Lahore, Pakistan (Riaz et al., 2012), but they reported a low percentage prevalence of *K. pneumoniae* from various clinical samples; however, pus samples in this study were also the least possible clinical source of *K. pneumoniae*. Another study reported from Karachi Pakistan revealed comparable findings regarding the percentage prevalence (17%) of *K. pneumoniae* from urine and blood samples (Khan et al., 2010a).

In the present study, about 70% of the *K. pneumoniae* clinical isolates were found to be ESBL-producing *K. pneumoniae*, which is a very alarming health situation prevailing in Pakistan. Isolates exhibited various antibiotic resistance determinants (ARDs) e.g., *bla*_*TEM*_ and *bla*_*CTX*–*M*_, which were associated with conferring the resistance against beta-lactam antibiotics. These findings were worrying because in local health settings most of the prescribed regimens are limited to a specific class of antibiotics. Same findings showing up to 70% ESBL producing *K. pneumoniae* have been reported by Shah et al. (2003), while another study reported 30% ESBL producing *K. pneumoniae* from Lahore (Ejaz et al., 2013). Comparable reports have been published from neighboring countries like India and Iran, where they found 56 and 59% ESBL producing *K. pneumoniae* from various samples, respectively (Jain and Mondal, 2007; Ghafourian et al., 2011). In a study from India, the prevalence of ESBL and carbapenemase-producing *K. pneumoniae* isolates were 47 and 17%, respectively (Wyres et al., 2020). In another study from India, 84% of *K. pneumoniae* isolates were ESBL producers whereas the carbapenem resistance phenotypes were observed in 66% of isolates (Bhaskar et al., 2019).

Additionally, a study conducted in the recent past found ESBL producing *K. pneumoniae* with a rate of 15.8% (Bukhari et al., 2016), the low percentage prevalence may be due to the small sampling fraction and a huge diversity of sample sources as compared to our studied samples. Worryingly, a significant percentage prevalence (32%) of carbapenem-resistant *K. pneumoniae* has also been detected in the present study. Isolates exhibited different ARGs which are responsible for the resistance against carbapenems e.g., *bla*_*KPC*_ (1.67%), *bla*_*OXA*_ (38.0%), and *bla*_*NDM*–1_ (43.3%), etc. It has been well characterized that *K. pneumoniae* displays carbapenem resistance by involving various mechanisms, like carbapenemases ARGs, outer membrane permeability alterations, and efflux pump system (Pitout et al., 2015). Few reports of carbapenem resistance in Pakistan have been published, and a recent study revealed the first blaOXA-181-producing carbapenem-resistant (CR) *K. pneumoniae* from Pakistan, which rang the alarm of pan-drug resistant (PDR) *K. pneumoniae* (Nahid et al., 2017). In a study from the largest tertiary care hospital in Bangladesh, the prevalence of carbapenem-resistant KP isolates was 30%. Most isolates were found to harbor *bla*_*NDM*–1_ i.e., 53% followed by *bla*_*NDM*–5_ (14%), *bla*_*OXA*–181_ (12%), and *bla*_*OXA*–232_ (10%) (Okanda et al., 2020).

From Pakistan, in livestock, there is barely any such report available to compare our findings. But the percentage prevalence estimated in the present study is closely relative to the findings of a study conducted in India, where they found a 6% percentage prevalence of Enterobacterales from various samples of veterinary sources, especially milk and fecal samples (Kar et al., 2015). The same sort of study has been conducted in England and Wales in 2011; they detected ESBLs-harboring bacteria in various samples of veterinary origin like fecal samples, farm premises samples, and waste milk (Randall et al., 2014). They also detected *bla*_*CTX*–*M*_ in waste milk, and they also suggested the key role of the farm environment in the dissemination of ARGs. A study has been reported from Germany, where they have detected the plasmid-mediated ESBLs producing gram-negative bacteria from cattle suffering from mastitis (Freitag et al., 2017). A significant prevalence of CR *K. pneumoniae* in poultry meat has been reported in China (Wu et al., 2016). Although they have identified a list of ARDs like the findings of the study, they have also reported *bla*_*CTX*–*M*_ as one of the most prevalent resistance genes in the samples. In a meta-analysis from India, the prevalence of ESBL-producing bacteria increased from 12% in 2013 to 33% in 2019 in veterinary settings. The species-wise prevalence of ESBL phenotypes was 5% in *Pseudomonas* spp., 9% in *E. coli*, and 10% in *K. pneumoniae*, which shows that the ESBL-producing *K. pneumoniae* is the leading ESBL-producing bacterial species in India (Kuralayanapalya et al., 2019).

Overall, a total of 21 discrete STs with their specific allelic pattern were identified from all the isolates. The most abundant ST found in the study was ST11; a total of 13 (70%) isolates were assigned with ST11, and among them 07 isolates were clinical specimens and 06 isolates were from veterinary samples. Overall, due to the unavailability of data on *K. pneumoniae* STs, we do not have such studies to corroborate our findings. In a retrospective study from Zhejiang, China from 2008 to 2018, it is reported that the prevalence of carbapenem-resistant *K. pneumoniae* increased from 2.5% (2008) to 15.8% (2018). Interestingly, the ST11 was the most common sequence type and blaKPC-2 was the most prevalent carbapenem-resistant determinant (Hu et al., 2020). In the recent past, sporadic reports of *K. pneumoniae* ST11 have been published from Pakistan (Qamar et al., 2018). In contrast to the findings which were specifically designed for *bla*_*NDM*–1_, the results of the present study revealed a comprehensive status of *K. pneumoniae* ST11 due to the molecular resistance pattern depicting various ARGs. The second most observed ST was the ST48; 12 isolates were allotted with the ST48, and among these isolates, 11 isolates belong to clinical origin while 01 sample was from an environmental source. ST11 and ST48 were found to be MDR as they showed a significant resistance pattern to almost all the antibiotics used in the study except colistin (Qamar et al., 2018).

To the best of our knowledge, this is the very first report describing the dissemination of *bla*_*KPC*–2_ producing *K. pneumoniae* ST258 at the human–environment interface from Pakistan. The global distribution of CG258 is well recognized and has been well reported. The findings of the study are partly in agreement with the available literature, as ST11, a group member of CG258 was the most abundant ST found in the study. Additionally, *bla*_*KPC*–2_ producing *K. pneumoniae* ST258 was also a novel finding from Pakistan. Globally, ST258 is known as a key etiology of MDR *K. pneumoniae*, especially carbapenemase-producing *K. pneumoniae* infections (Munoz-Price et al., 2013; Chen et al., 2014; Pitout et al., 2015).

## Conclusion

In conclusion, the major findings of the present study strengthened the understanding of the vital resistant determinants of *K. pneumoniae* which are never reported from Pakistan. Additionally, genetic diversity among indigenous *K. pneumoniae* isolates from different sources enabled us to find out different STs from humans, animals, and the associated environment, which may be very helpful in the future to control the public and animal health menace caused by MDR *K. pneumoniae*. Although it is one of the comprehensive studies reported from Pakistan, it also has some limitations. Particularly, the present study described the genetic diversity in MDR *K. pneumoniae* based on MLST, while the genetic characterization based on whole genome sequence (WGS) analysis is lacking. In the future, a detailed investigation based on WGS surveillance of MDR *K. pneumoniae* could be a potential work plan to decipher the genetic relatedness of this pathogen at the human–animal–environment interface.

## Data availability statement

The original contributions presented in this study are included in the article/supplementary material, further inquiries can be directed to the corresponding author/s.

## Ethics statement

The study was conducted in a span of 2 years from September 2017 to August 2019; proper permission was granted by the Ethical Review Board (ERB) of Government College University, Faisalabad, Pakistan.

## Author contributions

All authors listed have made a substantial, direct, and intellectual contribution to the work, and approved it for publication.

## Footnotes

1
https://bigsdb.pasteur.fr/klebsiella/
